# Applicability of the Framingham Risk Score in predicting mortality among kidney failure patients: an international analysis

**DOI:** 10.1093/ckj/sfaf296

**Published:** 2025-09-25

**Authors:** Zhimin Chen, Xiejia Li, Abdul Rashid Qureshi, Rending Wang, Hong Jiang, Olof Heimbürger, Peter Barany, Jianghua Chen, Peter Stenvinkel, Bengt Lindholm

**Affiliations:** Kidney Disease Center, 1st Affiliated Hospital College of Medicine, Zhejiang University, Hangzhou, China; Renal Medicine, Department of Clinical Sciences, Intervention and Technology, Karolinska Institutet, Stockholm, Sweden; Renal Medicine, Department of Clinical Sciences, Intervention and Technology, Karolinska Institutet, Stockholm, Sweden; Department of Nephrology, Second Xiangya Hospital, Central South University, Changsha, China; Renal Medicine, Department of Clinical Sciences, Intervention and Technology, Karolinska Institutet, Stockholm, Sweden; Kidney Disease Center, 1st Affiliated Hospital College of Medicine, Zhejiang University, Hangzhou, China; Kidney Disease Center, 1st Affiliated Hospital College of Medicine, Zhejiang University, Hangzhou, China; Renal Medicine, Department of Clinical Sciences, Intervention and Technology, Karolinska Institutet, Stockholm, Sweden; Renal Medicine, Department of Clinical Sciences, Intervention and Technology, Karolinska Institutet, Stockholm, Sweden; Kidney Disease Center, 1st Affiliated Hospital College of Medicine, Zhejiang University, Hangzhou, China; Renal Medicine, Department of Clinical Sciences, Intervention and Technology, Karolinska Institutet, Stockholm, Sweden; Renal Medicine, Department of Clinical Sciences, Intervention and Technology, Karolinska Institutet, Stockholm, Sweden

**Keywords:** cardiovascular disease, end-stage renal disease, Framingham Risk Score, mortality, traditional risk factors

## Abstract

**Background:**

The usefulness of the Framingham Risk Score (FRS) for estimation of mortality risk in kidney failure patients was explored in three cohorts of end-stage renal disease (ESRD) patients.

**Methods:**

Associations of the FRS with mortality risk were analysed among 392 Swedish non-dialysed (median age 56 years, males 62.8%, median FRS 19.5%) and 109 haemodialysed (median age 66 years, males 57.8%, median FRS 17.4%) ESRD patients and 1276 Chinese peritoneal dialysis (PD) patients (median age 50 years, males 55.6%, FRS 12.3%). We used Fine–Gray models to estimate relative risk associated with the FRS and its components, restricted mean survival time (RMST) to quantitate survival differences associated with the FRS and receiver operating characteristics curve analysis to estimate prognostic value of adding non-traditional risk factors to the FRS.

**Results:**

Despite different baseline characteristics, a 1 standard deviation increase in the FRS associated with similar increases (subhazard ratios 1.39–1.63) of both all-cause and cardiovascular mortality risk after adjusting for body mass index, serum albumin, haemoglobin and C-reactive protein (CRP). RMST and ∆RMST showed shorter survival time compared with all-cause and cardiovascular mortality in middle and high versus low FRS tertile. High versus low FRS tertiles showed 2- to 4-fold higher cumulative incident risk of all-cause and cardiovascular mortality. Adding non-traditional risk factors such as serum albumin and CRP to the FRS improved risk prediction marginally.

**Conclusions:**

A higher FRS associated with similar increases in mortality risk in three distinctly different cohorts of ESRD patients, whereas the addition of non-traditional risk factors had a negligible impact. The FRS is a valid tool for estimation of mortality risk in ESRD patients.

KEY LEARNING POINTS **What was known:**

- Cardiovascular disease (CVD) is the leading cause of death in end-stage renal disease (ESRD) patients.
- The Framingham Risk Score (FRS) is a valid tool for estimating mortality risk in general population.
- The usefulness of the FRS for estimation of mortality risk in kidney failure patients has been debated.

**This study adds:**

- This analysis showed that the FRS is a valid tool for estimation of mortality risk in three cohorts of ESRD patients from Sweden and China despite their differences in age, comorbidities, clinical and laboratory characteristics, treatment modality, ethnicity and geographical context.
- The results confirm that traditional risk factors are the main determinants of clinical outcomes in patients with kidney failure and that the addition of non-traditional risk factors to the FRS did improve risk prediction, but only marginally.
- Associations were to some extent context dependent and the lower impact of the FRS on risk prediction in prevalent haemodialysis (HD) patients suggests that a better understanding of reverse epidemiology and identification of relevant non-traditional risk factors may potentially help to improve risk prediction in this distinct population.

**Potential impact:**

- Although the FRS appears to be a valid tool for estimation of mortality risk in three cohorts of non-dialysed and dialysed ESRD patients from China and Sweden, the FRS had a limited impact on risk prediction in prevalent HD patients, suggesting the need for a deeper understanding of reverse epidemiology and the identification of relevant non-traditional risk factors to enhance risk prediction in this population.
- Further research into reverse epidemiology and non-traditional risk factors could improve risk prediction models for ESRD patients, particularly those on HD.

## INTRODUCTION

Chronic kidney disease (CKD) is associated with high morbidity and mortality [1, 2]. Cardiovascular disease (CVD) is the main cause of mortality [3] and accounts for ≈40–50% of all deaths in patients with end-stage renal disease (ESRD) [4]. The excessive cardiovascular risk is mainly attributed to the high prevalence of traditional risk factors [e.g. hypertension and diabetes mellitus (DM)] among people with CKD, although non-traditional risk factors (e.g. inflammation and oxidative stress) also play a significant role [5, 6].

The Framingham Risk Score (FRS) estimates the 10-year cardiovascular risk of an individual in the general population based on traditional cardiovascular risk factors: age, sex, systolic blood pressure (SBP), treatment of hypertension, total cholesterol (TC), high-density lipoprotein cholesterol (HDL-C), DM and smoking status [7]. The usefulness of the FRS for estimation of mortality risk in kidney failure patients has been debated. The FRS was reported to predict cardiovascular event probability in Spanish CKD stage 1–4 patients [8] and all-cause and cardiovascular mortality risk in Swedish CKD stage 5 patients [9], whereas in Chinese haemodialysis (HD) patients, intermediate risk categorized by the FRS, but not high risk, predicted mortality [10]. In other studies in CKD patients, the FRS was a rather poor predictor of outcomes [11, 12].

One reason for these differences could be that traditional risk factors in CKD patients may differ from those in the general population [13, 14]. Furthermore, the FRS does not include components specific to the atherogenic uraemic milieu [15, 16]. The addition of CKD-specific variables such as kidney function and albuminuria to the FRS was suggested to improve the predictive value of the FRS in such patients [17], but some studies failed to show improvement in the predictive power of the FRS by adding CKD-specific variables [11, 12]. In contrast, cardiovascular risk factors such as obesity, hypercholesterolaemia and hypertension were reported to be protective and associated with greater survival in maintenance dialysis patients, a phenomenon called reverse epidemiology [13].

In the present study, we evaluated the association of the FRS with the risk of all-cause and cardiovascular mortality in three distinctly different cohorts of ESRD patients: Swedish non-dialysed (ND) CKD stage 5 patients, Swedish haemodialysis (HD) patients and Chinese patients undergoing peritoneal dialysis (PD). The aims of the study were to determine the extent to which traditional cardiovascular mortality risk factors, represented by the FRS, associate with mortality risk in ESRD; establish whether the association of the FRS with mortality risk in ESRD patients is robust or context dependent; and explore if the addition of non-traditional risk factors such as C-reactive protein (CRP) and albumin to the FRS may increase its utility as a mortality risk predictor in ESRD.

## MATERIALS AND METHODS

### Patients and study design

We investigated FRS in a total of 1777 ESRD patients: 392 Swedish ND patients were recruited from the malnutrition, inflammation and atherosclerosis (MIA) cohort study of incident dialysis patients [18], 109 Swedish HD patients were recruited from the Mapping of Inflammatory Markers in Chronic Kidney Disease studies on prevalent HD (MIMICK1) [19, 20] and 1276 Chinese incident PD patients were recruited from a longitudinal cohort study at the Department of Kidney Disease Centre, First Affiliated Hospital College of Medicine, Zhejiang University, Hangzhou, China [21].

All patients were followed until renal transplantation or death or until completing 60 months of follow-up. Time to renal transplantation or death and cause of death (cardiac or non-cardiac reason) were documented. The local ethics committees approved study protocols. Studies adhered to the Declaration of Helsinki. Informed written consent was obtained from each participant.

### Measurements of clinical and laboratory data

Biochemical and clinical parameters of patients were analysed as previously described [18, 19, 21].

### Endpoints and outcome measures

Cardiovascular mortality was defined as death caused by coronary events, arrhythmia, sudden cardiac death, cardiac failure or cerebrovascular accident. All other causes of death were designated as non-cardiovascular mortality. Cause of death was established by the death certificate issued by the attending physician.

### Assessment of the FRS

The FRS, an estimate of the 10-year risk of developing CVD, for each patient was calculated as a sex-specific risk score incorporating age, smoking status, DM, SBP, anti-hypertensive medication, TC and HDL-C [7].

### Statistical analysis

Continuous variables are presented as median [interquartile range (IQR)]. Categorical variables are presented as number and percentage. Statistical significance was set at *P* < .05. Comparisons between two groups were assessed with the non-parametric Wilcoxon test for continuous variables and chi-squared test for nominal variables. Associations between the FRS and mortality were analysed with the FRS as a continuous variable using flexible parametric models yielding spline curves. Fine–Gray analysis with renal transplantation as a competing risk was performed to establish cumulative incidence curves and to provide risk estimates for patients in the middle and high tertiles of the FRS expressed as subhazard ratios (sHRs) with patients in the low FRS tertile serving as the reference. The risk score was then used to calculate the predictive validity of each model using receiver operating characteristics (ROC) curve analysis to calculate the ROC area under the curve (AUC). We further analysed the adjusted restricted mean survival time (RMST) with inverse probability of treatment weighting (IPTW) and expressed the results as the difference (∆RMST) between the tertiles of the FRS at disparate time points. Statistical analyses were performed using Stata 18 (Stat Corp, College Station, TX, USA) and SAS 9.4 M7 (SAS Institute, Cary, NC, USA).

## RESULTS

### Clinical and biochemical characteristics

Patients in the Swedish cohorts were older, more often males, with a higher prevalence of DM and current smoking and higher TC, HDL-C and SBP levels and thus had higher FRSs [18.6% (IQR 8.7–31.9) versus 12.3% (IQR 5.3–24.8), *P* < .001] compared with the Chinese cohort. Also, most risk factors associated with CVD and mortality were significantly different between the Swedish and Chinese cohorts (Supplementary Table 1).

In 501 Swedish patients, 180 patients (35.9%) died and 230 (41.1%) underwent renal transplantation during a median 2.2 years of follow-up and 78 (43.3%) of the 180 deaths were caused by CVD. Among 392 Swedish ND patients, 116 (25.9%) died and 201 (51.2%) underwent renal transplantation; 51 (44%) of the 116 deaths were caused by CVD. Among 109 Swedish HD patients, 64 (58.7%) died and 29 (26.6%) underwent renal transplantation; 27 (42.1%) of the 64 deaths were caused by CVD. In 1276 Chinese patients, 199 (15.6%) died and 324 (25.4%) patients underwent renal transplantation during a median 3.7 years of follow-up; 91 (45.7%) of the 199 deaths were caused by CVD.

The clinical and biochemical characteristics—overall and categorized by FRS tertiles—are shown in Table 1. In the Swedish cohorts, among 392 ND patients (median age 56 years, males 62.8%, median FRS 19.5%), patients with high-tertile FRSs had a higher prevalence of CVD, higher body mass index (BMI) and high-sensitivity CRP (hsCRP) and lower serum albumin, whereas these differences between tertiles of the FRS were absent or weaker among 109 HD patients (median age 66 years, males 57.8%, median FRS 17.4%). In the Chinese cohort (median age 50 years, males 55.6%, median FRS 12.3%), patients with high-tertile FRSs had a higher prevalence of CVD, BMI, hsCRP and haemoglobin and lower serum albumin, phosphate and intact parathyroid hormone (iPTH).

**Table 1:** Clinical and biochemical characteristics among three cohorts of ESRD patients: (**A**) ND and (**B**) HD patients from Sweden and (**C**) PD patients from China for all patients in each cohort and when divided according to the FRS.

(A) Swedish ND patients	All (*N* = 392)	Low FRS tertile (*n* = 131)	Middle FRS tertile (*n* = 131)	High FRS tertile (*n* = 130)	*P*-value
Age (years)	56.0 (46.0–65.0)	41.0 (32.0–50.0)	58.0 (50.0–65.0)	65.0 (59.0–69.0)	<.001
Male, *n* (%)	246 (62.8)	56 (42.7)	84 (64.1)	106 (81.5)	<.001
Diabetes, *n* (%)	119 (30.4)	16 (12.2)	41 (31.3)	62 (47.7)	<.001
CVD, *n* (%)	142 (36.2)	12 (9.2)	54 (41.2)	76 (58.5)	<.001
Current smoker, *n* (%)	55 (14.0)	11 (8.4)	19 (14.5)	25 (19.2)	.041
Anti-hypertension medication, *n* (%)	360 (91.8)	114 (87.0)	121 (92.4)	125 (96.2)	.026
SBP (mmHg)	150.0 (136.0–165.0)	140.0 (126.0–151.0)	147.0 (136.0–162.0)	165.0 (150.0–179.0)	<.001
BMI (kg/m^2^)	24.4 (22.0–28.4)	23.1 (20.4–26.7)	24.5 (22.3–28.4)	25.7 (22.9–28.8)	<.001
hsCRP (mg/l) (*n* = 391)	4.3 (1.4–13.0)	2.7 (1.0–9.1)	4.7 (1.5–13.4)	6.8 (2.4–17.0)	<.001
IL-6 (pg/ml)	6.3 (3.6–10.8)	4.4 (2.1–7.5)	6.5 (3.7–12.7)	7.7 (4.6–11.8)	<.001
Albumin (g/l)	33.0 (30.0–36.0)	35.0 (31.0–38.0)	33.0 (30.0–36.0)	33.0 (29.0–35.0)	<.001
Triglyceride (mmol/l) (*n* = 391)	1.7 (1.2–2.2)	1.4 (1.0–2.0)	1.7 (1.2–2.2)	1.9 (1.4–2.6)	<.001
LDL-C (mmol/l) (*n* = 390)	2.4 (1.7–3.2)	2.1 (1.6–2.8)	2.3 (1.9–3.2)	2.9 (1.9–3.8)	<.001
HDL-C (mmol/l) (*n* = 391)	1.2 (0.9–1.5)	1.4 (1.0–1.6)	1.2 (0.9–1.6)	1.1 (0.8–1.4)	<.001
TC (mmol/l) (*n* = 391)	4.5 (3.8–5.4)	4.4 (3.6–5.1)	4.5 (3.8–5.4)	4.8 (3.8–5.9)	.021
iPTH (ng/l) (*n* = 389)	239.0 (126.0–377.0)	233.0 (126.0–381.0)	223.0 (113.0–395.0)	246.5 (150.9–348.0)	.84
Calcium (mmol/l) (*n* = 389)	2.4 (2.2–2.6)	2.4 (2.2–2.5)	2.4 (2.2–2.6)	2.4 (2.2–2.6)	.43
Phosphate (mmol/l) (*n* = 388)	1.9 (1.6–2.3)	2.0 (1.6–2.4)	1.9 (1.6–2.3)	1.9 (1.6–2.3)	.40
Haemoglobin (g/l)	106.0 (96.0–115.0)	107.0 (95.0–115.0)	108.0 (98.0–116.0)	104.0 (95.0–112.0)	.15
FRS (%)	19.5 (8.2–32.1)	5.2 (2.9–8.3)	19.5 (15.3–23.5)	41.6 (32.2–56.7)	<.001
(B) Swedish HD patients	All (*N* = 109)	Low tertile (*n* = 37)	Middle tertile (*n* = 36)	High tertile (*n* = 36)	*P*-value
Age (years)	66.0 (51.0–75.0)	53.0 (43.0–70.0)	65.5 (52.5–74.5)	72.5 (64.0–78.5)	<.001
Male, *n* (%)	63 (57.8)	16 (43.2)	21 (58.3)	26 (72.2)	.043
Diabetes, *n* (%)	26 (23.9)	4 (10.8)	7 (19.4)	15 (41.7)	.006
CVD, *n* (%)	67 (61.5)	17 (45.9)	25 (69.4)	25 (69.4)	.058
Current smoker, *n* (%)	23 (21.1)	6 (16.2)	8 (22.2)	9 (25.0)	.64
Anti-hypertension medication, *n* (%)	65 (59.6)	13 (35.1)	25 (69.4)	27 (75.0)	<.001
SBP (mmHg)	140.0 (130.0–160.0)	135.0 (120.0–140.0)	140.0 (132.5–162.5)	150.0 (140.0–170.0)	<.001
BMI (kg/m^2^) (*n* = 103)	23.1 (20.2–26.9)	23.6 (20.4–27.1)	22.9 (20.1–26.0)	22.7 (20.7–27.6)	.97
hsCRP (mg/l) (*n* = 108)	8.9 (2.7–24.0)	12.5 (3.0–29.0)	7.1 (2.9–21.0)	10.0 (2.5–20.5)	.78
IL-6 (pg/ml)	9.3 (5.5–15.6)	8.6 (4.4–15.4)	8.2 (4.4–18.0)	10.0 (8.4–17.4)	.17
Albumin (g/l)	34.0 (32.0–36.0)	34.0 (31.0–38.0)	34.0 (32.0–36.0)	34.0 (32.0–36.0)	.78
Triglyceride (mmol/l) (*n* = 108)	1.6 (1.1–2.1)	1.4 (1.0–2.3)	1.6 (1.1–1.9)	1.6 (1.1–2.2)	.69
LDL-C (mmol/l) (*n* = 108)	2.1 (1.5–2.5)	1.7 (1.0–2.2)	2.0 (1.7–2.4)	2.3 (1.7–3.0)	.014
HDL-C (mmol/l) (*n* = 108)	1.3 (1.1–1.8)	1.4 (1.2–1.9)	1.5 (1.2–1.9)	1.2 (1.0–1.5)	.050
TC (mmol/l) (*n* = 108)	4.3 (3.7–5.0)	4.2 (3.4–4.5)	4.4 (3.7–5.0)	4.4 (3.9–5.1)	.23
iPTH (ng/l)	/	/	/	/	
Calcium (mmol/l)	2.6 (2.5–2.7)	2.5 (2.4–2.7)	2.6 (2.5–2.7)	2.5 (2.4–2.6)	.31
Phosphate (mmol/l)	1.8 (1.5–2.1)	1.7 (1.4–2.1)	1.6 (1.5–2.2)	1.9 (1.5–2.2)	.69
Haemoglobin (g/l)	120.0 (110.0–126.0)	121.0 (110.0–127.0)	115.0 (104.0–126.0)	119.0 (112.5–125.5)	.42
FRS (%)	17.4 (9.9–29.7)	8.3 (4.3–9.9)	17.5 (14.3–20.7)	37.2 (30.5–45.9)	<.001
(C) Chinese PD patients	All (*N* = 1276)	Low tertile (*n* = 426)	Middle tertile (*n* = 425)	High tertile (*n* = 425)	*P*-value
Age (years)	50.0 (40.0–60.0)	38.0 (30.0–44.0)	51.0 (44.0–58.0)	61.0 (53.0–69.0)	<.001
Male, *n* (%)	709 (55.6)	152 (35.7)	229 (53.9)	328 (77.2)	<.001
Diabetes, *n* (%)	176 (13.8)	5 (1.2)	37 (8.7)	134 (31.5)	<.001
CVD, *n* (%)	82 (6.4)	14 (3.3)	19 (4.5)	49 (11.5)	<.001
Current smoker, *n* (%)	305 (23.9)	19 (4.5)	84 (19.8)	202 (47.5)	<.001
Anti-hypertension medication, *n* (%)	1160 (90.9)	376 (88.3)	384 (90.4)	400 (94.1)	.011
SBP (mmHg)	145.0 (134.0–156.0)	140.0 (126.0–147.0)	145.0 (134.0–154.0)	150.0 (144.0–168.0)	<.001
BMI (kg/m^2^)	21.2 (19.3–23.3)	20.4 (18.5–22.1)	21.2 (19.4–23.3)	22.1 (20.2–24.4)	<.001
hsCRP (mg/l) (*n* = 936)	1.8 (0.0–5.3)	1.4 (0.0–2.9)	1.8 (0.0–6.4)	2.6 (0.0–7.1)	<.001
Albumin (g/l)	36.8 (33.3–40.5)	38.3 (34.3–41.5)	37.1 (34.1–40.7)	35.8 (31.7–39.0)	<.001
Triglyceride (mmol/l)	1.4 (1.0–1.9)	1.3 (0.9–1.7)	1.4 (1.0–1.9)	1.5 (1.1–2.0)	.002
LDL-C (mmol/l)	2.2 (1.8–2.8)	2.2 (1.8–2.6)	2.2 (1.8–2.7)	2.4 (1.9–3.0)	<.001
HDL-C (mmol/l)	1.0 (0.9–1.3)	1.1 (0.9–1.3)	1.0 (0.9–1.3)	1.0 (0.8–1.2)	<.001
TC (mmol/l)	4.2 (3.6–4.9)	4.1 (3.5–4.7)	4.1 (3.5–4.9)	4.4 (3.7–5.2)	<.001
iPTH (ng/l)	304.5 (164.5–454.0)	329.5 (196.0–526.0)	329.5 (181.0–466.0)	254.0 (132.0–371.0)	<.001
Calcium (mmol/l)	2.1 (2.0–2.2)	2.1 (2.0–2.2)	2.1 (2.0–2.2)	2.1 (1.9–2.2)	.024
Phosphate (mmol/l)	1.8 (1.5–2.1)	1.8 (1.6–2.2)	1.8 (1.5–2.0)	1.8 (1.5–2.0)	<.001
Haemoglobin (g/l)	82.0 (72.0–93.0)	80.0 (70.0–89.0)	82.0 (70.0–95.0)	84.7 (74.0–94.0)	<.001
FRS (%)	12.3 (5.3–24.8)	3.8 (2.5–5.3)	12.3 (9.7–15.2)	33.9 (24.8–48.8)	<.001

Values are presented as median (IQR) unless stated otherwise.

### Association of the FRS with all-cause and cardiovascular mortality

Risk estimates (sHR) for the association of a 1-SD increase in the FRS with all-cause and cardiovascular mortality after adjusting for BMI, serum albumin, haemoglobin and hsCRP are shown in Table 2. The association of the FRS with mortality was significant in the Swedish ND patients [sHR 1.49 (95% CI 1.26–1.76), *P* < .001 for all-cause mortality and sHR 1.47 (95% CI 1.14–1.90), *P* = .003 for cardiovascular mortality], as well as in the PD patients from China [sHR 1.49 (95% CI 1.30–1.70), *P* < .001 for all-cause mortality and sHR 1.63 (95% CI 1.35–1.97), *P* < 0.001 for cardiovascular mortality]. In HD patients from Sweden, the association of the FRS with mortality was significant for all-cause mortality risk [HR 1.39 (95% CI 1.08–1.79), *P* = .011], whereas the association with cardiovascular mortality was not significant [HR1.44 (95% CI 0.97–2.13), *P* = .068]. An increase of albumin was associated with decreased all-cause mortality risk in all three cohorts. An increase of BMI was associated with decreased all-cause mortality and an increase in hsCRP was associated with higher cardiovascular mortality in Swedish ND patients.

**Table 2:** Risk of all-cause and cardiovascular mortality risk associated with a 1-SD increase of the FRS and other risk markers in three cohorts of ESRD patients.

	All-cause mortality			Cardiovascular mortality		
392 Swedish ND patients	sHR	95% CI	*P*-value	sHR	95% CI	*P*-value
1-SD increase of FRS (%)	**1.49**	1.26–1.76	**<.001**	**1.47**	1.14–1.90	**.003**
1-SD increase of BMI (kg/m^2^)	0.77	0.63–0.95	**.013**	0.80	0.59–1.08	.150
1-SD increase of albumin (g/l)	0.76	0.62–0.94	**.010**	0.92	0.67–1.26	.619
1-SD increase of haemoglobin (g/l)	1.09	0.90–1.31	.381	1.03	0.78–1.35	.817
1-SD increase of hsCRP (mg/l)	1.08	0.93–1.27	.299	1.24	1.05–1.46	**.011**
109 Swedish HD patients	sHR	95% CI	*P*-value	sHR	95% CI	*P*-value
1-SD increase of FRS (%)	**1.39**	1.08–1.79	**.011**	1.44	0.97–2.13	.068
1-SD increase of BMI (kg/m^2^)	0.76	0.57–1.02	.064	0.85	0.55–1.29	.436
1-SD increase of albumin (g/l)	0.64	0.42–0.98	**.041**	0.80	0.43–1.51	.497
1-SD increase of haemoglobin (g/l)	0.89	0.69–1.13	.331	0.77	0.51–1.15	.200
1-SD increase of hsCRP (mg/l)	0.99	0.74–1.33	.949	1.00	0.63–1.59	.990
1276 Chinese PD patients	sHR	95% CI	*P*-value	sHR	95% CI	*P*-value
1-SD increase of FRS (%)	**1.49**	1.31–1.70	**<.001**	**1.63**	1.35–1.97	**<.001**
1-SD increase of BMI (kg/m^2^)	0.91	0.76–1.08	.280	1.08	0.84–1.39	.536
1-SD increase of albumin (g/l)	0.72	0.60–0.86	**<.001**	0.83	0.64–1.08	.161
1-SD increase of haemoglobin (g/l)	1.01	0.85–1.21	.875	1.07	0.83–1.38	.622
1-SD increase of hsCRP (mg/l) (*n* = 936)	1.02	0.90–1.16	.719	0.97	0.76–1.25	.839

Significant values in bold.

**Figure. 1:**
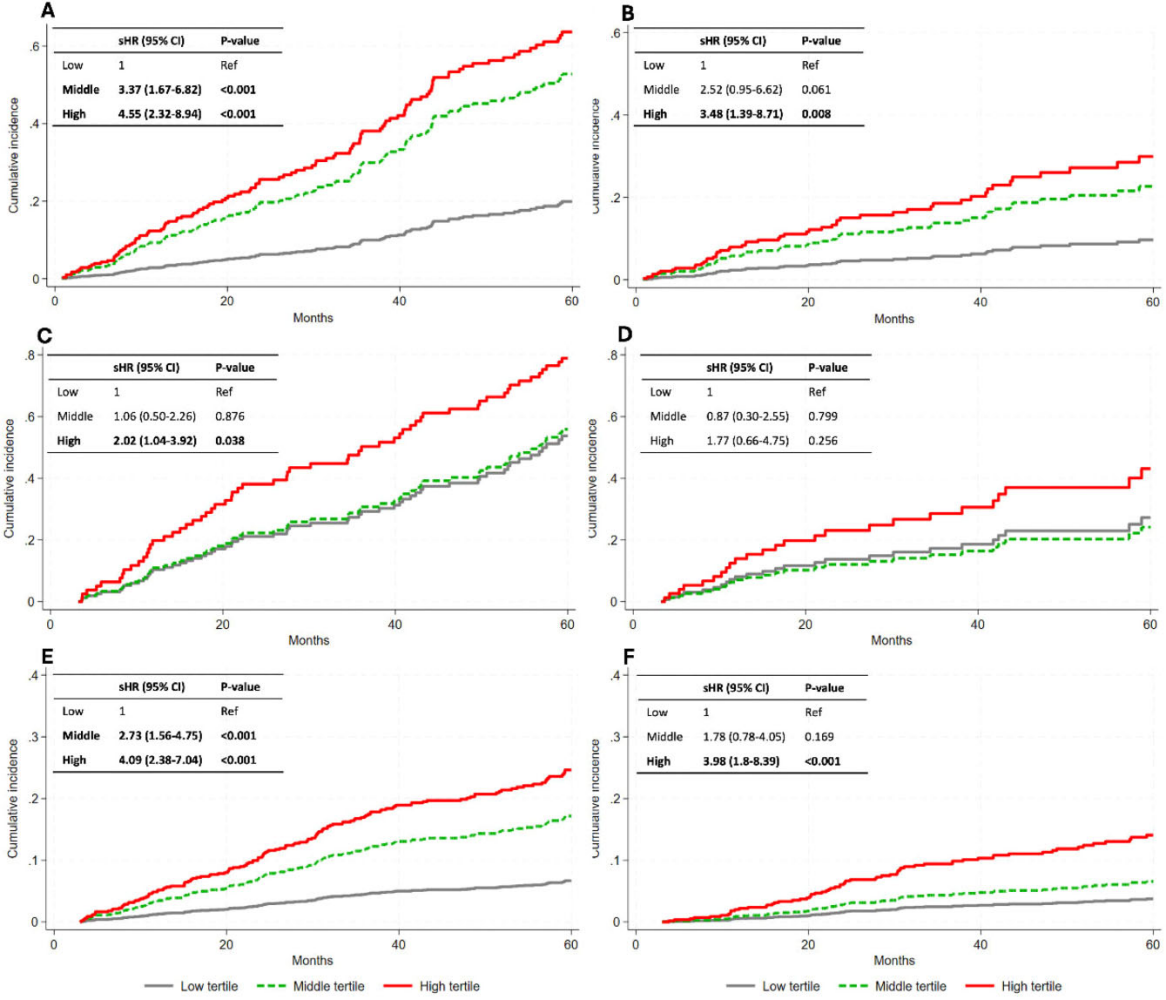
Fine–Gray competing risk analysis of cumulative incidence of mortality with inserts showing sHRs and 95% confidence intervals for all-cause mortality in **(A)** 392 Swedish ND, **(C)** 109 Swedish HD and **(E)** 1276 Chinese PD patients and cardiovascular mortality in **(B)** 392 Swedish ND, **(D)** 109 Swedish HD and **(F)** 1276 Chinese PD patients for tertiles of the FRS after adjusting for BMI, serum albumin, haemoglobin and hsCRP.

Fine–Gray analysis taking renal transplantation as a competing risk showed that the cumulative incident risk of all-cause mortality was significantly higher in middle and high FRS tertiles than in the low tertile after adjustments, in both ND patients (Fig. 1A) and PD patients (Fig. 1E). The corresponding associations of the FRS tertiles with cardiovascular mortality were significant in high tertile in ND (Fig. 1B) and PD (Fig. 1F) patients. In Swedish HD patients, a high tertile FRS was significantly associated with the cumulative incident risk of all-cause mortality (Fig. 1C) but not with cardiovascular mortality (Fig. 1D).

**Figure 2:**
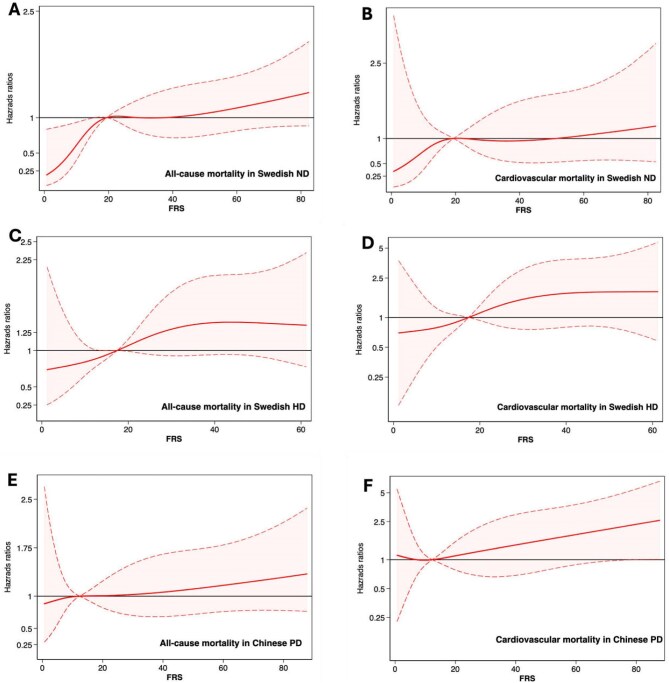
Spline curves showing multivariate HRs (95% confidence intervals) for the FRS after adjusting for BMI, serum albumin, haemoglobin and hsCRP of all-cause mortality in **(A)** Swedish ND, **(C)** Swedish HD and **(E)** Chinese PD patients and cardiovascular mortality in **(B)** Swedish ND, **(D)** Swedish HD and **(F)** Chinese PD patients.

Analysis by spline curves showing HRs for all-cause and cardiovascular mortality after adjusting for BMI, serum albumin, haemoglobin and hsCRP in ND, HD and PD patients confirmed that a higher FRS was associated with higher all-cause and cardiovascular mortality (Fig. 2).

### RMST in relation to FRS tertiles

**Figure 3:**
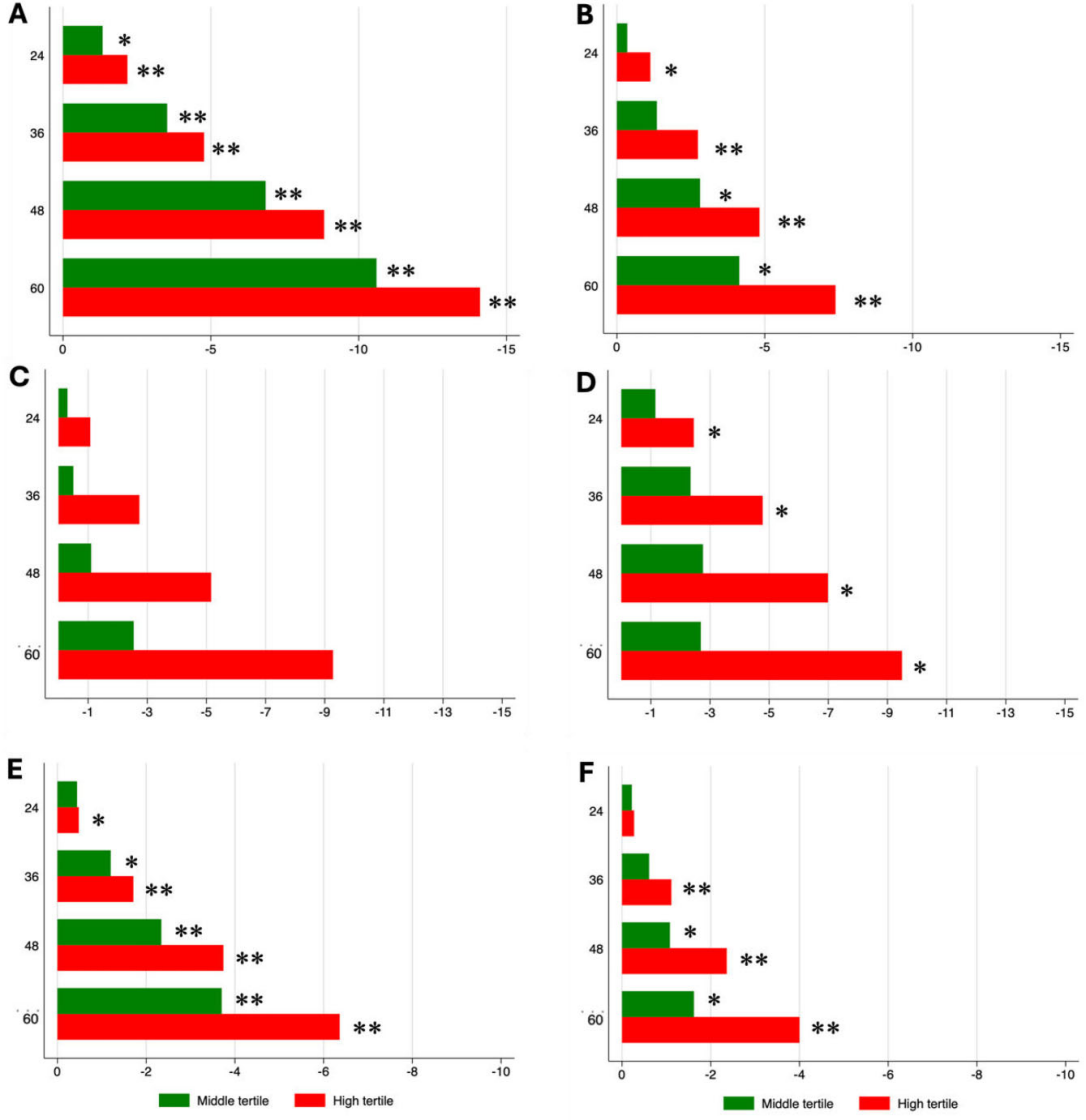
Shorter RMST (months) to all-cause mortality in **(A)** Swedish ND, **(C)** Swedish HD and **(E)** Chinese PD patients and to the event of cardiovascular mortality in **(B)** Swedish ND, **(D)** Swedish HD and **(F)** Chinese PD patients at 24, 36, 48 and 60 months for middle and high tertiles compared with the low tertile of the FRS. *P < .05, **P < .01.

The RMST to events of all-cause and cardiovascular mortality at 24, 36, 48 and 60 months, after adjusting for BMI, serum albumin, haemoglobin and calendar year, was with few exceptions significantly shorter for middle and high FRS tertiles versus the low tertile, i.e. mortality occurred on average significantly earlier in those with a higher FRS (Supplementary Table 2). The corresponding differences (ΔRMST) until all-cause and cardiovascular mortality occurred are provided in Fig. 3 and Supplementary Table 2, which shows for example that at follow-up of 60 months, Swedish ND patients within the high FRS tertile died on average 14.1 months earlier than those in the low FRS tertile.

### ROC analysis of adding biomarkers to the FRS model

**Figure 4:**
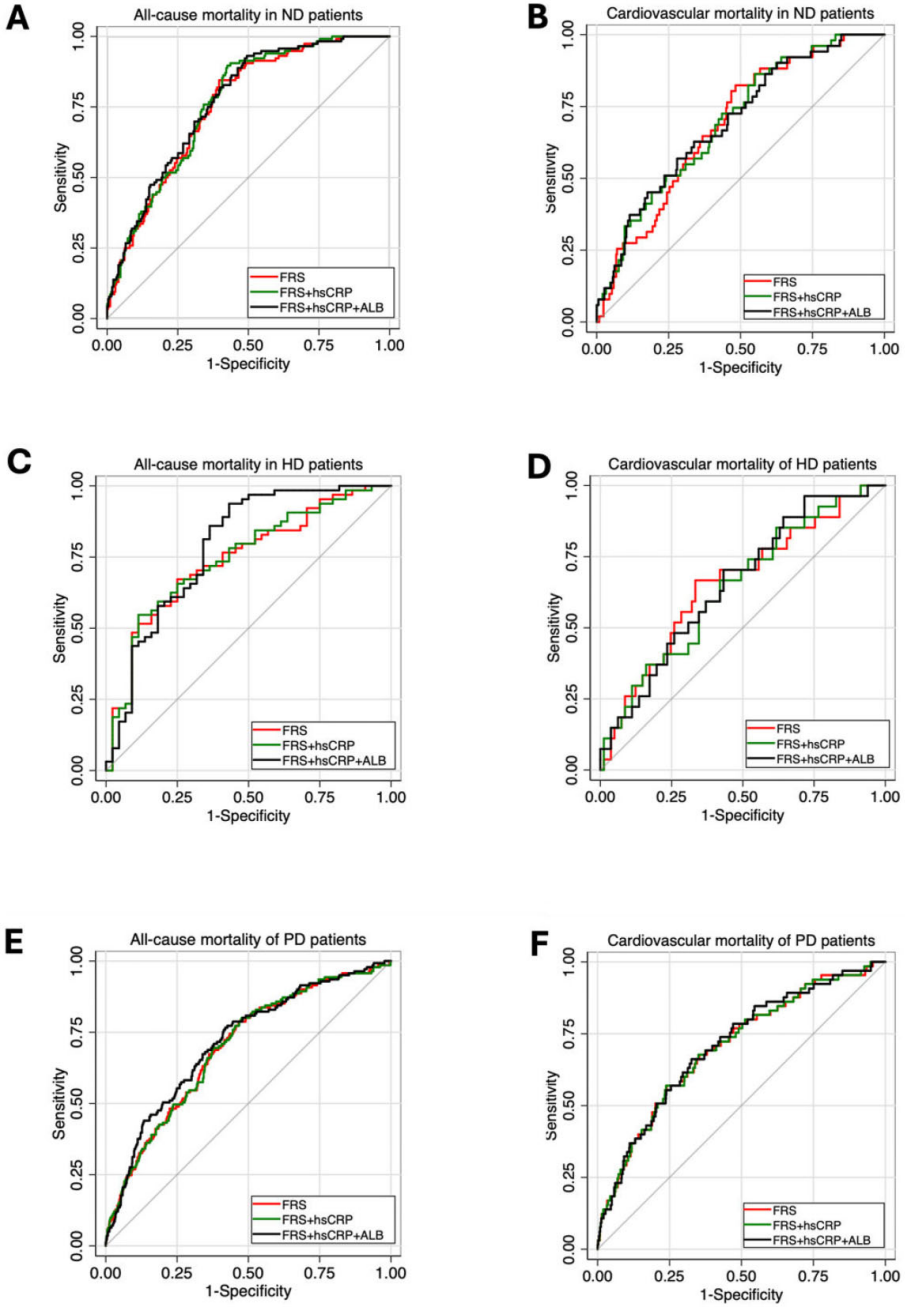
ROC-AUCs describing the ability of the FRS with and without adding hsCRP and albumin (ALB) to predict all-cause and cardiovascular mortality in **(A, B)** in Swedish ND patients, **(C, D)** in Swedish HD patients and **(E, F)** in Chinese PD patients.

To evaluate the extent to which adding non-traditional risk factors to the FRS improves the prediction of mortality, we calculated the ROC-AUC for the FRS with and without hsCRP and albumin, respectively. The results showed some improvements of discriminatory performance by adding hsCRP and albumin to the FRS (Fig. 4). We have reported that circulating interleukin-6 (IL-6) was a reliable predictor of mortality risk in Swedish ESRD patients [22]. Therefore, we calculated the ROC-AUC for the FRS with and without IL-6 and albumin, respectively, for the prediction of all-cause and cardiovascular mortality in Swedish ND and HD patients, while IL-6 was not available in the Chinese PD patients. However, the results did not show a major improvement in the predictive power by adding IL-6 and albumin to the FRS (Supplementary Table 3). Adding CKD-specific factors such as phosphate, calcium and iPTH also did not substantially improve the predictive power of the FRS (Supplementary Table 4).

### Association of FRS components with mortality

As reverse epidemiology of cardiovascular risk factors was reported to play a role in maintenance dialysis patients [13], we further investigated the impact of the components of the FRS on mortality (Table 3). As expected, older age and the presence of DM were significantly associated with increased all-cause and cardiovascular mortality risk in Swedish ND patients and Chinese PD patients. In Swedish HD patients, older age and current smoking were associated with significantly increased all-cause mortality risk, while a 1-SD increase in SBP was associated with lower all-cause mortality risk [sHR 0.67 (95% CI 0.53–0.85)] as well as with lower cardiovascular risk [HR 0.64 (95% CI 0.45–0.92)].

**Table 3:** Multivariate Fine–Gray competing risk analysis of all-cause and cardiovascular mortality associated with components of the FRS after adjusting for BMI, serum albumin and haemoglobin.

	All-cause mortality			Cardiovascular mortality		
Cohort	sHR	95% CI	*P*-value	sHR	95% CI	*P*-value
392 Swedish ND patients: 1-SD increase of FRS	**1.49**	1.26–1.76	**<.001**	**1.47**	1.14–1.90	**.003**
Components of FRS						
1-SD increase of age (year)	2.55	1.88–3.45	<.001	2.61	1.64–4.14	<.001
Sex (male versus female)	1.17	0.80–1.73	.414	1.09	0.61–1.96	.761
Presence of DM (no = 0, yes = 1)	1.91	1.27–2.87	.002	2.93	1.59–5.40	.001
1-SD increase of TC (mmol/l)	1.02	0.85–1.23	.825	0.86	0.63–1.16	.323
1-SD increase of HDL-C (mmol/l)	1.19	1.04–1.36	.014	1.25	1.06–1.47	.009
1-SD increase of SBP (mmHg)	0.81	0.66–0.99	.037	0.94	0.70–1.25	.658
Anti-hypertensive medication use (no = 0, yes = 1)	1.23	0.53–2.85	.635	2.07	0.49–8.70	.322
Current smoker (no = 0, yes = 1)	1.24	0.77–2.00	.377	1.41	0.69–2.85	.343
109 Swedish HD patients: 1-SD increase of FRS	**1.39**	1.08–1.79	**.011**	1.44	0.97–2.13	.068
Components of FRS						
1-SD increase of age (year)	1.83	1.30–2.58	.001	1.3	0.82–2.15	.248
Sex (male versus female)	1.02	0.60–1.74	.945	1.35	0.57–3.17	.495
Presence of DM (no = 0, yes = 1)	1.53	0.85–2.77	.157	1.96	0.82–4.69	.131
1-SD increase of TC (mmol/l)	0.95	0.73–1.25	.731	0.92	0.61–1.40	.705
1-SD increase of HDL-C (mmol/l)	0.95	0.72–1.26	.724	1.01	0.66–1.54	.963
1-SD increase of SBP (mmHg)	0.67	0.53–0.85	.001	0.64	0.45–0.92	.017
Anti-hypertensive medication use (no = 0, yes = 1)	1.43	0.82–2.50	.204	3.31	1.18–9.29	.023
Current smoker (no = 0, yes = 1)	2.18	1.15–4.13	.017	2.67	1.05–6.82	.04
1276 Chinese PD patients: 1-SD increase of FRS	**1.49**	1.31–1.70	**<.001**	**1.63**	1.35–1.97	**<.001**
Components of FRS						
1-SD increase of age (year)	2.18	1.82–2.60	<.001	2.08	1.60–2.71	<.001
Sex (male versus female)	0.78	0.56–1.10	.152	0.94	0.57–1.56	.823
Presence of DM (no = 0, yes = 1)	1.42	1.16–1.74	.001	1.84	1.40–2.42	<.001
1-SD increase of TC (mmol/l)	1.03	0.93–1.14	.542	1.06	0.93–1.20	.389
1-SD increase of HDL-C (mmol/l)	0.98	0.82–1.16	.802	0.96	0.74–1.24	.754
1-SD increase of SBP (mmHg)	1.15	0.98–1.36	.087	1.20	0.95–1.52	.12
Anti-hypertensive medication use (no = 0, yes = 1)	0.33	0.19–0.56	<.001	0.42	0.17–1.08	.072
Current smoker (no = 0, yes = 1)	0.68	0.44–1.04	.073	0.72	0.39–1.33	.297

Significant values in bold.

## DISCUSSION

In this cross-country analysis, higher FRS was associated with increased mortality risk in three cohorts of ESRD patients from China and Sweden despite their differences in age, comorbidities, clinical and laboratory characteristics, treatment modality, ethnicity and geographical context. The decisive impact of traditional risk factors represented by the FRS on the prognosis of ESRD patients is further underlined by our finding that there were only negligible improvements in the predictive power when non-traditional risk factors were added to the FRS.

The usefulness of the FRS as a predictor of cardiovascular risk is well established in the general population, while there are inconsistent findings regarding the association of the FRS with outcomes in CKD patients [6–10]. Studies in ND CKD patients showed that the FRS can predict cardiovascular events [6, 19] as well as all-cause and cardiovascular mortality risk in ESRD patients [8]. While FRS might underestimate coronary events (including myocardial infarction and coronary death) in individuals with CKD by 2- to 3-fold [23], there is evidence that the FRS can predict cardiovascular events in this high-risk population [8, 24]

In our study, a higher FRS was associated with worse survival in three diverse cohorts of ESRD patients, and spline curves revealed positive linear relationships between the FRS and the risk of all-cause and cardiovascular mortality. Moreover, beyond these hazard function analyses, survival function analysis using the RMST model provided clinically relevant quantitative estimates of survival among each tertile of the FRS. Risk estimates such as HR or sHR are measures of relative risk that may not reflect either survival time or survival rate. In some cases, even if the absolute effect of different factors on survival time is small, the HR or sHR can be misleadingly high. Therefore, measures of relative risk should be considered together with other indicators, such as median survival time or survival rate at a given time point. RMST analysis provides a quantitative measure of survival time among each group at a given time point, which offers a more realistic and clinically meaningful estimate of survival than HR or sHR. In our study, RMST analysis showed that patients with a higher FRS (middle and high FRS tertile versus low tertile) had a shorter survival time; the time to occurrence of all-cause and cardiovascular mortality events at a follow-up time of 24, 36, 48 and 58 months was decreased. However, in the Chinese PD cohort, differences in the RMST (i.e. ∆RMST) between tertiles were not significant at 24 months, suggesting that it may take time for the effects linked to the FRS to show up as increased mortality and that the follow-up period should be long enough to demonstrate a clear difference.

The FRS was created using data from a population-based cohort in which the majority was not burdened by CKD or other non-traditional risk factors, while in patients with renal insufficiency, non-traditional factors such as inflammation, malnutrition and anaemia are prevalent and might increase the risk of CVD and poor outcomes [15, 16]. Therefore, the addition of specific variables to the components of the FRS was suggested to improve the predictive value of the FRS in such patients [17]. Inflammation, a common feature in ESRD [25], plays a pivotal role in processes leading to atherosclerosis [26], and associates with CVD [27], cardiovascular events [28] and poor outcome [18]. In the present study, we analysed the ROC-AUC for the FRS with and without hsCRP and albumin for the prediction of all-cause and cardiovascular mortality risk but found only negligible improvements of discrimination. As IL-6 may be a more reliable biomarker than hsCRP as a predictor of CVD and mortality risk [22, 29], we calculated the ROC-AUC for the FRS with and without adding IL-6 and albumin; however, this did not improve the prediction of all-cause and cardiovascular mortality risk in ESRD patients. Other studies also failed to show improvement in the predictive power of the FRS by adding CKD-specific variables [11, 17, 30].

Good progress has been made so far in predicting cardiovascular events and mortality in CKD patients using novel risk factors, from a single score integrating multiple biomarkers [31, 32] and cardiovascular risk prediction models incorporating multiple traditional and novel risk factors [33, 34] to the utilization of a targeted proteomics assay [35], and several of these risk-predicting methods displayed great performance. However, some predictive models are derived from intricate logistic or proportional hazards models and include multiple variables, some of which are not commonly available in routine clinical practice. The FRS combines important traditional risk factors of CVD in a systematic way, and these factors are usually accessible in routine clinical practice based on clinical assessment, routine biochemical examinations and medical history and have the potential for modification. Therefore, the FRS might be a simple, easily available and useful tool for evaluating outcomes in the ESRD population, although it is to some extent context dependent.

In our study, the cohort of Swedish HD patients differed from the other two cohorts in that the FRS (and only the high tertile) associated with all-cause mortality risk but not with cardiovascular mortality risk. We speculate that one explanation could be the time discrepancy between competitive risk factors. For example, survival disadvantages of undernutrition, which is frequently present in dialysis patients, may contribute to mortality in the short term, and this could overwhelm negative long-term effects of overnutrition on survival [13]. Swedish patients were followed up for a median 2.2 years, while Chinese PD patients were followed up for a median of 3.7 years. Second, survival bias may play a role since only a small number of patients with CKD survive long enough to reach ESRD. Hence, dialysis patients—and especially prevalent patients—represent a distinctively selected population of survivors and may not represent the risk factor constellations of their CKD predecessors.

In the present study, we also found that higher SBP in the Swedish HD patients was associated with a decreased mortality risk. This is in line with the observation that conventional risk factors such as obesity, hypercholesterolaemia and hypertension may appear to be protective features among dialysis patients [13].

In the present study, we found similar associations between the FRS and death risk in Chinese and Swedish ND ESRD patients despite marked differences in baseline characteristics, including the FRS and its components, and a much lower crude mortality rate among the younger Chinese patients. Although the FRS and mortality rate differed between the cohorts, the relevance of a higher FRS as a predictor of increased mortality risk, expressed as multivariate adjusted HR, was similar.

The present study has some limitations and strengths that should be considered when interpreting the results. First, this is an observational study, and no conclusions can be made about causality. Second, we did not analyse coronary heart disease over 10 years and measured risk indicators only at a single time point at baseline. Indeed, a higher incidence of cardiovascular events in the first weeks [36] and increased mortality during the first 3 months after HD initiation have been observed [37]. It is plausible that dynamic assessments could improve the accuracy compared with measurements at a single time point. Third, several covariates were not explored, such as proteinuria, coronary artery calcification score, residual renal function, dialysis ultrafiltration rate and peritoneal dialysate glucose exposure. Finally, there were many significant differences in basic patient characteristics between the three cohorts of patients (Table 1). For example, the Chinese PD patients were younger, DM and CVD were less common and BMI was lower than in the two Swedish cohorts (Supplementary Table 1). We cannot exclude that differences are due to different diagnostic procedures or reflect differences in healthcare systems and health status between China and Sweden [38, 39]. Despite these differences, the FRS appears to be a valid predictor of mortality risk in the investigated cohorts from the two countries.

Strengths include careful follow-up of a relatively large number of patients, although the results need to be confirmed in larger cohorts and in other ethnic populations. Furthermore, the impact of the FRS was described not only in terms of relative risk but also by analysis of the RMST for the FRS tertiles, which may be more informative and intuitive to clinical communities as these measures describe quantitative differences of estimated survival time, expressed as the number of months over defined follow-up periods.

In conclusion, this study shows that the FRS is a valid tool for estimation of mortality risk in ESRD. Thus a higher FRS was associated with an increased all-cause and cardiovascular mortality risk in three distinctly diverse cohorts of ESRD patients in two countries despite differences in age, comorbidities, clinical and laboratory characteristics, treatment modality, ethnicity and geographical context. The results confirm that traditional risk factors are the main determinants of clinical outcomes in patients with kidney failure and that the addition of non-traditional risk factors to the FRS did improve risk prediction only marginally. However, associations were to some extent context-dependent and the lower impact of the FRS on risk prediction in prevalent HD patients suggests that a better understanding of reverse epidemiology and identification of relevant non-traditional risk factors may potentially help to improve risk prediction in this distinct population.

## Supplementary Material

sfaf296_Supplemental_File

## Data Availability

The data underlying this article are available in the article and in its online supplementary material.
