# Supplementary material for: Applicability of the Framingham Risk Score in predicting mortality among kidney failure patients: an international analysis
Source: Clin Kidney J. 2025 Sep 25;18(11):sfaf296. doi: 10.1093/ckj/sfaf296 (PMC12598281; doi:10.1093/ckj/sfaf296)
Supplement: sfaf296_Supplemental_File [file sfaf296_supplemental_file.docx]

| **Variables** | **Sweden** | **China** | **p-value** |
| --- | --- | --- | --- |
|  | N=501 | N=1276 |  |
| Age, years | 58.0 (47.0-67.0) | 50.0 (40.0-60.0) | <0.001 |
| Male, n (%) | 309 (61.7%) | 709 (55.6%) | 0.019 |
| Diabetes, n (%) | 145 (28.9%) | 176 (13.8%) | <0.001 |
| CVD, n (%) | 209 (41.7%) | 82 (6.4%) | <0.001 |
| Current smoking, n (%) | 78 (15.6%) | 305 (23.9%) | <0.001 |
| Anti-hypertension medication, n (%) | 425 (84.8%) | 1,160 (90.9%) | <0.001 |
| Systolic blood pressure, mmHg | 149.0 (135.0-165.0) | 145.0 (134.0-156.0) | 0.002 |
| BMI, kg/m^2^ | 24.1 (21.8-28.1) | 21.2 (19.3-23.3) | <0.001 |
| hsCRP, mg/L | 5.4 (1.7-14.1) | 1.8 (0.0-5.3)^#^ | <0.001 |
| Albumin, g/L | 34.0 (30.0-36.0) | 36.8 (33.3-40.5) | <0.001 |
| Triglyceride, mmol/L | 1.6 (1.1-2.2) | 1.4 (1.0-1.9) | <0.001 |
| LDL-C, mmol/L | 2.3 (1.7-3.1) | 2.2 (1.8-2.8) | 0.68 |
| HDL-C, mmol/L | 1.2 (1.0-1.6) | 1.0 (0.9-1.3) | <0.001 |
| Total cholesterol, mmol/L | 4.5 (3.8-5.3) | 4.2 (3.6-4.9) | <0.001 |
| iPTH, ng/L | 239.0 (126.0-377.0) | 304.5 (164.5-454.0) | <0.001 |
| Calcium, (mmol/L) | 2.4 (2.2-2.6) | 2.1 (2.0-2.2) | <0.001 |
| Phosphate, mmol/L | 1.9 (1.6-2.3) | 1.8 (1.5-2.1) | <0.001 |
| Hemoglobin, g/L | 109.0 (98.0-118.0) | 82.0 (72.0-93.0) | <0.001 |
| Framingham`s risk score, % | 18.6 (8.7-31.9) | 12.3 (5.3-24.8) | <0.001 |

**Supplementary Table 1.** Comparison of the clinical and biochemical characteristics in the Swedish and Chinese cohorts.

Continuous variables are presented as median (25th – 75th percentile). Categorical variables are presented as number (n)/percentage (%). Abbreviations: CVD, Cardiovascular disease; BMI, body mass index; HDL-C, high-density lipoprotein cholesterol; iPTH, intact parathyroid hormone; hsCRP, high-sensitivity C-reactive protein. ^#^ n=936

**Supplementary Table 2.** Restricted mean survival time (RMST) and differences in RMST (∆RMST) for all-cause and cardiovascular mortality at 24, 36, 48 and 60 months among 382 Swedish non-dialyzed (ND) ESRD patients, 109 Swedish hemodialysis (HD) patients and 1276 Chinese peritoneal dialysis (PD) patients.

Statistically significant differences are marked: ^*^ P<0.05; ^**^ P<0.01.

|  | | RMST (months) | | | ∆RMST (SE) (months) | |
| --- | --- | --- | --- | --- | --- | --- |
|  | | Low tertile | Middle tertile | High tertile | Middle vs. Low | High vs. Low |
| **ND patients** All-cause mortality | 24 | 23.09 | 21.75 | 20.91 | -1.34 (0.49)^*^ | -2.18 (0.55)^**^ |
|  | 36 | 34.19 | 30.67 | 29.42 | -3.52 (0.91)^**^ | -4.77 (0.98)^**^ |
|  | 48 | 44.79 | 37.94 | 35.96 | -6.85 (1.39)^**^ | -8.83 (1.41)^**^ |
|  | 60 | 54.86 | 44.26 | 40.78 | -10.6 (1.95)^**^ | -14.1 (1.85)^**^ |
| **ND patients** Cardiovascular mortality | 24 | 23.23 | 22.88 | 22.10 | -0.35 (0.37) | -1.13 (0.46)^*^ |
|  | 36 | 34.71 | 33.36 | 31.97 | -1.35 (0.71) | -2.74 (0.85)^**^ |
|  | 48 | 45.97 | 43.17 | 41.16 | -2.81 (1.14)^*^ | -4.82 (1.27)^**^ |
|  | 60 | 56.90 | 52.76 | 49.51 | -4.14 (1.65)^*^ | -7.39 (1.77)^**^ |
| **HD patients** All-cause mortality | 24 | 21.07 | 20.77 | 20.00 | -0.30 (1.03) | -1.07 (1.05) |
|  | 36 | 29.47 | 28.97 | 26.74 | -0.50 (1.83) | -2.73 (1.87) |
|  | 48 | 37.01 | 35.91 | 31.86 | -1.10 (2.65) | -5.16 (2.65) |
|  | 60 | 44.17 | 41.63 | 34.89 | -2.54 (3.45) | -9.28 (3.22) |
| **HD patients** Cardiovascular mortality | 24 | 23.08 | 21.94 | 20.64 | -1.15 (0.85) | -2.45 (1.04)^*^ |
|  | 36 | 33.93 | 31.60 | 29.15 | -2.34 (1.59) | -4.78 (1.88)^*^ |
|  | 48 | 43.80 | 41.04 | 36.82 | -2.76 (2.40) | -6.99 (2.74)^*^ |
|  | 60 | 53.18 | 50.49 | 43.69 | -2.69 (3.23) | -9.49 (3.64)^*^ |
| **PD patients** All-cause mortality | 24 | 23.37 | 22.92 | 22.89 | -0.44 (0.19) | -0.48 (0.19)^*^ |
|  | 36 | 34.65 | 33.46 | 32.94 | -1.20 (0.37)^*^ | -1.71 (0.37)^**^ |
|  | 48 | 45.84 | 43.49 | 42.09 | -2.34 (0.57)^**^ | -3.74 (0.59)^**^ |
|  | 60 | 56.92 | 53.22 | 50.56 | -3.70 (0.80)^**^ | -6.36 (0.85)^**^ |
| **PD patients** Cardiovascular mortality | 24 | 23.74 | 23.52 | 23.47 | -0.22 (0.13) | -0.27 (0.13) |
|  | 36 | 35.44 | 34.83 | 34.33 | -0.61 (0.26) | -1.11 (0.28)^**^ |
|  | 48 | 47.09 | 46.01 | 44.74 | -1.08 (0.41)^*^ | -2.36 (0.47)^**^ |
|  | 60 | 58.72 | 57.10 | 54.71 | -1.62 (0.57)^*^ | -4.00 (0.69)^**^ |

**Supplementary Table 3.** Areas under the ROC curve (AUC) describing the ability of Framingham Risk Score (FRS) with or without adding high-sensitivity C-reactive protein (hsCRP) and albumin (ALB) to predict all-cause and cardiovascular mortality in ESRD patients.

| **Model** | **All-cause mortality** | | | | **Cardiovascular mortality** | | | |
| --- | --- | --- | --- | --- | --- | --- | --- | --- |
| 392 ND patients | AUC | | 95% CI | | AUC | | 95% CI | |
| **FRS** | 0.763 | 0.712 | | 0.814 | 0.696 | 0.625 | | 0.768 |
| **FRS + hsCRP** | 0.770 | 0.721 | | 0.820 | 0.702 | 0.629 | | 0.775 |
| **FRS + hsCRP+ALB** | 0.775 | 0.726 | | 0.825 | 0.701 | 0.626 | | 0.776 |
| **FRS + IL6** | 0.778 | 0.729 | | 0.827 | 0.716 | 0.647 | | 0.786 |
| **FRS + IL6+ALB** | 0.780 | 0.730 | | 0.828 | 0.714 | 0.642 | | 0.786 |
| 109 HD patients |  |  | |  |  |  | |  |
| **FRS** | 0.742 | 0.646 | | 0.836 | 0.656 | 0.535 | | 0.777 |
| **FRS + hsCRP** | 0.746 | 0.652 | | 0.841 | 0.644 | 0.525 | | 0.763 |
| **FRS + hsCRP+ALB** | 0.788 | 0.695 | | 0.881 | 0.655 | 0.540 | | 0.771 |
| **FRS + IL6** | 0.786 | 0.696 | | 0.876 | 0.655 | 0.538 | | 0.772 |
| **FRS + IL6+ALB** | 0.799 | 0.710 | | 0.888 | 0.654 | 0.541 | | 0.768 |
| 1276 PD patients |  |  | |  |  |  | |  |
| **FRS** | 0.696 | 0.649 | | 0.742 | 0.703 | 0.637 | | 0.771 |
| **FRS + hsCRP** | 0.697 | 0.650 | | 0.742 | 0.703 | 0.636 | | 0.770 |
| **FRS + hsCRP+ALB** | 0.715 | 0.669 | | 0.760 | 0.708 | 0.642 | | 0.774 |

**Supplementary Table 4.** Areas under the ROC curve (AUC) describing the ability of Framingham Risk Score (FRS) to predict all-cause and cardiovascular mortality in ESRD patients when adding CKD-specific factors such as phosphate, calcium, and iPTH in addition to high-sensitivity C-reactive protein (hsCRP) and albumin (ALB).

| **Model** | **All-cause mortality** | | | | **Cardiovascular mortality** | | | |
| --- | --- | --- | --- | --- | --- | --- | --- | --- |
| 392 ND patients | AUC | | 95% CI | | AUC | | 95% CI | |
| **FRS** | 0.763 | 0.712 | | 0.814 | 0.696 | 0.625 | | 0.768 |
| **FRS + hsCRP** | 0.770 | 0.721 | | 0.820 | 0.702 | 0.629 | | 0.775 |
| **FRS + hsCRP+ALB** | 0.775 | 0.726 | | 0.825 | 0.701 | 0.626 | | 0.776 |
| **FRS + IL6** | 0.778 | 0.729 | | 0.827 | 0.716 | 0.647 | | 0.786 |
| **FRS + IL6+ALB** | 0.780 | 0.730 | | 0.828 | 0.714 | 0.642 | | 0.786 |
| **FRS + hsCRP+ALB+Ca** | 0.771 | 0.724 | | 0.819 | 0.701 | 0.628 | | 0.774 |
| **FRS + hsCRP+ALB+Ca+P** | 0.773 | 0.726 | | 0.821 | 0.702 | 0.628 | | 0.776 |
| **FRS + hsCRP+ALB+Ca+P+PTH** | 0.773 | 0.726 | | 0.821 | 0.701 | 0.628 | | 0.775 |
| 109 HD patients |  |  | |  |  |  | |  |
| **FRS** | 0.742 | 0.646 | | 0.836 | 0.656 | 0.535 | | 0.777 |
| **FRS + hsCRP** | 0.746 | 0.652 | | 0.841 | 0.644 | 0.525 | | 0.763 |
| **FRS + hsCRP+ALB** | 0.788 | 0.695 | | 0.881 | 0.655 | 0.540 | | 0.771 |
| **FRS + IL6** | 0.786 | 0.696 | | 0.876 | 0.655 | 0.538 | | 0.772 |
| **FRS + IL6+ALB** | 0.799 | 0.710 | | 0.888 | 0.654 | 0.541 | | 0.768 |
| **FRS + hsCRP+ALB+Ca** | 0.799 | 0.710 | | 0.887 | 0.672 | 0.548 | | 0.795 |
| **FRS + hsCRP+ALB+Ca+P** | 0.802 | 0.714 | | 0.890 | 0.670 | 0.547 | | 0.793 |
| 1276 PD patients |  |  | |  |  |  | |  |
| **FRS** | 0.696 | 0.649 | | 0.742 | 0.703 | 0.637 | | 0.771 |
| **FRS + hsCRP** | 0.697 | 0.650 | | 0.742 | 0.703 | 0.636 | | 0.770 |
| **FRS + hsCRP+ALB** | 0.715 | 0.669 | | 0.760 | 0.708 | 0.642 | | 0.774 |
| **FRS + hsCRP+ALB+Ca** | 0.716 | 0.671 | | 0.762 | 0.708 | 0.642 | | 0.774 |
| **FRS + hsCRP+ALB+Ca+P** | 0.728 | 0.683 | | 0.773 | 0.720 | 0.657 | | 0.783 |
| **FRS + hsCRP+ALB+Ca+P+PTH** | 0.728 | 0.683 | | 0.773 | 0.720 | 0.659 | | 0.782 |
